# Infertility as a possible diagnostic feature of Carney complex

**DOI:** 10.1210/jcemcr/luag065

**Published:** 2026-05-05

**Authors:** Pablo Knoblovits, Erika Abelleira, Gastón Rey Valzacchi, Sebastián Matias Suárez, Pablo René Costanzo, Soledad Kleppe

**Affiliations:** Servicio de Endocrinología, Metabolismo y Medicina Nuclear, Hospital Italiano de Buenos Aires, Ciudad Autónoma de Buenos Aires C1199ABB, Argentina; División Endocrinología, Hospital de Clínicas “José de San Martín”, Universidad de Buenos Aires, Ciudad Autónoma de Buenos Aires C1120AAF, Argentina; Servicio de Urología, Hospital Italiano de Buenos Aires, Ciudad Autónoma de Buenos Aires C1199ABB, Argentina; Servicio de Endocrinología, Metabolismo y Medicina Nuclear, Hospital Italiano de Buenos Aires, Ciudad Autónoma de Buenos Aires C1199ABB, Argentina; Servicio de Endocrinología, Metabolismo y Medicina Nuclear, Hospital Italiano de Buenos Aires, Ciudad Autónoma de Buenos Aires C1199ABB, Argentina; Genética y Enfermedades Metabólicas, Hospital Italiano de Buenos Aires, Ciudad Autónoma de Buenos Aires C1199ABB, Argentina

**Keywords:** Carney complex, azoospermia, infertility, testicular microlithiasis

## Abstract

Carney complex (CNC) is a rare autosomal dominant syndrome characterized by multiple neoplasms, including endocrine and testicular tumors. Infertility in men with CNC has been rarely reported and remains poorly understood. We describe a 39-year-old male with azoospermia and testicular microlithiasis as the sole manifestations of CNC, without other phenotypic features. Family history revealed CNC-related conditions in his mother and brother, and genetic testing confirmed a pathogenic *PRKAR1A* variant (c.479del; p.Ala160Glufs*5). Testicular biopsy showed hypospermatogenesis with premature spermatocyte and spermatid detachment, consistent with protein kinase A hyperactivation-related apoptosis. Intracytoplasmic sperm injection with retrieved sperm resulted in poor embryonic development, and the couple pursued donor sperm-assisted reproduction. Testicular microlithiasis is a recognized feature of CNC, but its role in male reproductive dysfunction remains unclear. Recent studies suggest *PRKAR1A* haploinsufficiency may impair spermatogenesis, leading to reduced fertility. Our findings align with reports of abnormal spermatogenesis in CNC-associated azoospermia. Male infertility may represent an underrecognized feature of CNC. This case highlights the importance of genetic evaluation in infertile men, particularly when testicular microlithiasis and a suggestive family history are present, to enable early recognition of CNC and ensure appropriate surveillance, counseling, and fertility management.

## Introduction

Carney complex (CNC) is a genetic multiple neoplasia syndrome characterized by pigmentary abnormalities of the skin and mucosa, cardiac and noncardiac myxomatous tumors, adrenocortical primary hyperpigmented micronodular hyperplasia, pituitary and other endocrine tumors. CNC is inherited in an autosomal dominant manner with high penetrance and variable expression. Familial transmission has been reported more frequently via an affected mother, suggesting a non-Mendelian inheritance pattern or impaired male fertility in affected individuals [1]. Nearly two-thirds of CNC patients are heterozygous for inactivating mutations in the gene encoding the protein kinase A (PKA) type I regulatory subunit, *PRKAR1A* [2] leading to constitutive, cAMP-independent activation of PKA signaling. Approximately 25% of cases occur sporadically as a result of a de novo mutation. Involvement in CNC may include testicular lesions as large cell calcifying sertoli cell tumors (LCCSCT) and other testicular tumors such as Leydig cell and adrenocortical rest tumors, which occur concurrently with LCCSCT [3]. LCCSCT or characteristic calcification on testicular ultrasound are considered as one of the major diagnostic criteria of CNC [4]. We report the case of a male with infertility and testicular microcalcifications as the sole manifestation of CNC.

## Case presentation

A 39-year-old Caucasian man was referred to the andrology unit of our department for evaluation of azoospermia, identified during the investigation of a 2-year history of infertility. His past medical history was unremarkable. He reported preserved libido and normal sexual function, with no history of pregnancies with previous partners. Testicular microcalcifications had been detected on ultrasound performed as follow-up for nonspecific testicular pain. The patient had been taking finasteride 1 mg daily for androgenic alopecia. Although azoospermia is not a commonly reported adverse effect of this medication, treatment was discontinued; however, repeat semen analysis several months later again demonstrated azoospermia. He worked in an administrative position with no known occupational or environmental exposures.

On physical examination, his weight was 84 kg and height 1.83 m. Blood pressure was normal, and there were no clinical features of hypercortisolism, acromegaly, or gynecomastia. Cutaneous manifestations suggestive of CNC were absent. Testicular volume, measured with a Prader orchidometer, was 25 mL bilaterally, and the vas deferens were palpable.

Hormonal evaluation showed luteinizing hormone 8.4 IU/L (reference range, 1.7-8.6 IU/L), follicle-stimulating hormone (FSH) 6.9 IU/L (reference range, 1.5-12.4 IU/L), total testosterone 458 ng/dL (SI: 15.88 nmol/L) (reference range, >280 ng/dL [SI: >9.7 nmol/L]), free testosterone 104.6 pg/mL (SI: 362.7 pmol/L) (reference range, 66-174 pg/mL [SI: 228.8-603.3 pmol/L]), insulin-like growth factor 1 229 ng/mL (SI: 29.9 nmol/L) (reference range, 57-241 ng/mL [SI: 7.4–31.5 nmol/L]), prolactin 9.4 ng/mL (SI: 9.4 μg/L) (reference range, <18 ng/mL [SI: <18 μg/L]), urinary free cortisol 48 ug/24 hours (SI: 132 nmol/24 hours) (reference range 4.3–176 μg/24 hours [SI: 11.9–485.8 nmol/24 hours]) and adrenocorticotropic hormone 36.8 pg/mL (SI: 8.10 pmol/L (reference range: 7.2–63.3 pg/mL [SI: 1.6–14 pmol/L]). Karyotype was normal, and no microdeletions in the Y-chromosome azoospermia factor were detected. Two semen analyses revealed volumes of 4.0 and 3.5 mL with pH values of 8.0 and 7.8, respectively; no sperm were identified in either sample, including after centrifugation.

**Figure 1:**
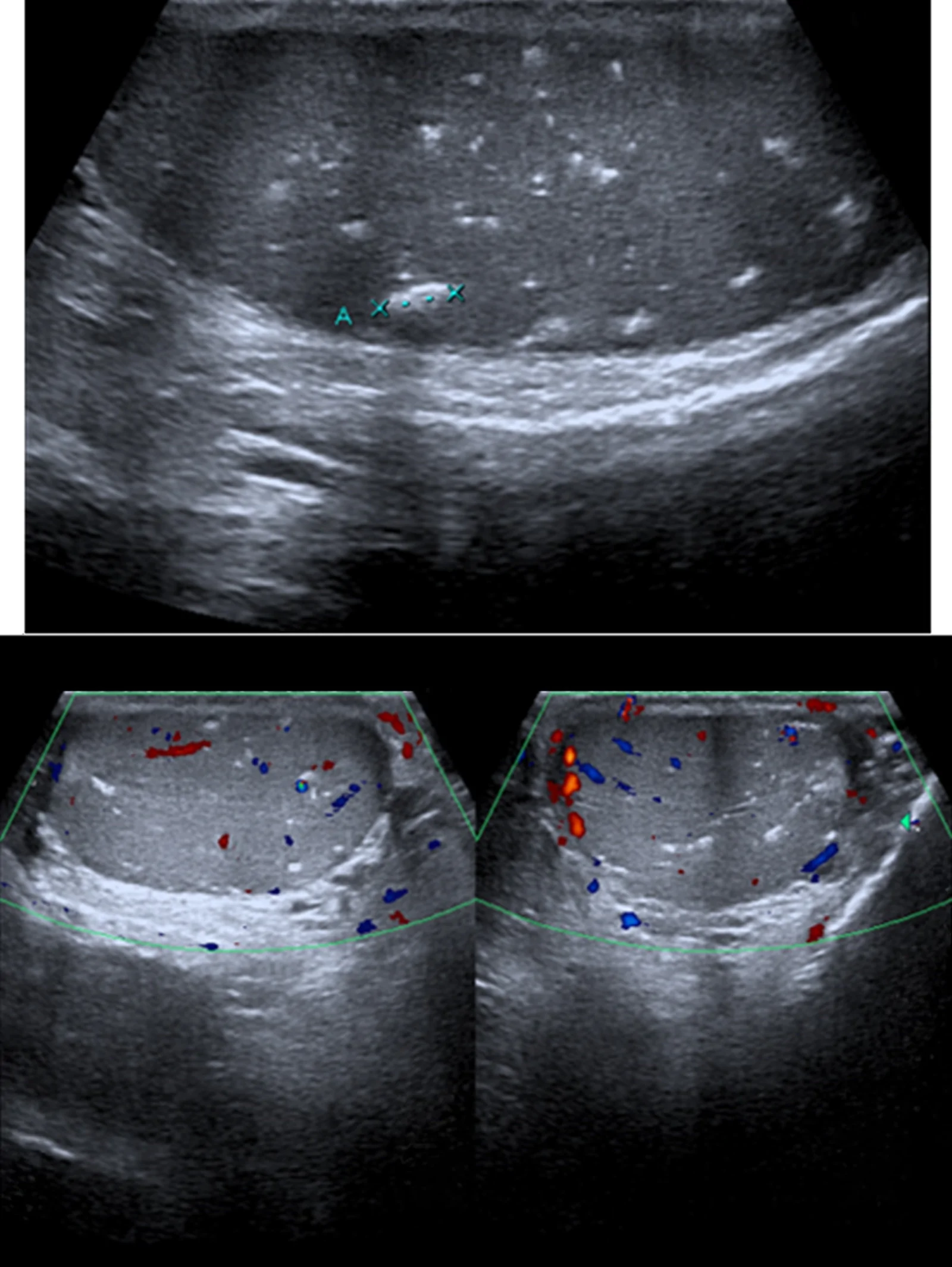
Testicular ultrasound showing microlithiasis.

Testicular ultrasound demonstrated both testes of preserved shape and size, but with heterogeneous echotexture due to multiple bilateral calcifications (4-6 mm on the right and 4 mm on the left), without focal lesions. Ultrasound-derived testicular volumes were 18.8 cm³ (right) and 14.4 cm³ (left) (Fig. 1). Transthoracic echocardiography, abdominal magnetic resonance imaging (MRI), and pituitary MRI showed no abnormalities. The patient's wife, aged 33 years, had normal fertility studies.

Family history was remarkable: his mother had undergone surgery for cardiac myxoma, presented lentigines, and developed benign breast nodules requiring mastectomy. His brother had an orchiectomy at age 8 for a sertoli cell tumor and later surgery for bilateral gynecomastia. He also had a history of a nonfunctioning, nonadenomatous right adrenal lesion and multiple pituitary cystic lesions (hypointense on T1 and T2 sequences) in the right lateral anterior and posterior sectors, with the largest left laterobasal lesion measuring 10 mm. In addition, the brother developed a pericardial effusion requiring surgical drainage.

## Diagnostic assessment

The findings were consistent with testicular azoospermia due to hypospermatogenesis and spermatogenic arrest, later confirmed by biopsy.

Molecular testing of the patient's mother, performed by sequencing and deletion/duplication analysis of the *PRKAR1A* gene, identified a pathogenic variant, c.479del (p.Ala160Glufs*5). Subsequent testing of the patient confirmed the presence of the same variant, consistent with autosomal dominant inheritance. The predicted frameshift introduces a premature stop codon expected to trigger nonsense-mediated mRNA decay and result in loss of normal RI-α protein expression and function. Loss-of-function mutations in *PRKAR1A* (including nonsense, frameshift, splice-site, and large deletions) are well-established causes of CNC, leading to haploinsufficiency of the RI-α subunit, increased PKA activity, and dysregulated cAMP signaling.

This specific variant has an entry in ClinVar, where it is classified as pathogenic, consistent with its truncating nature and the recognized mechanism of disease. To date, no publications describing this exact variant were identified in the literature, supporting its designation as a novel pathogenic allele in the context of *PRKAR1A* related disease.

**Figure 2:**
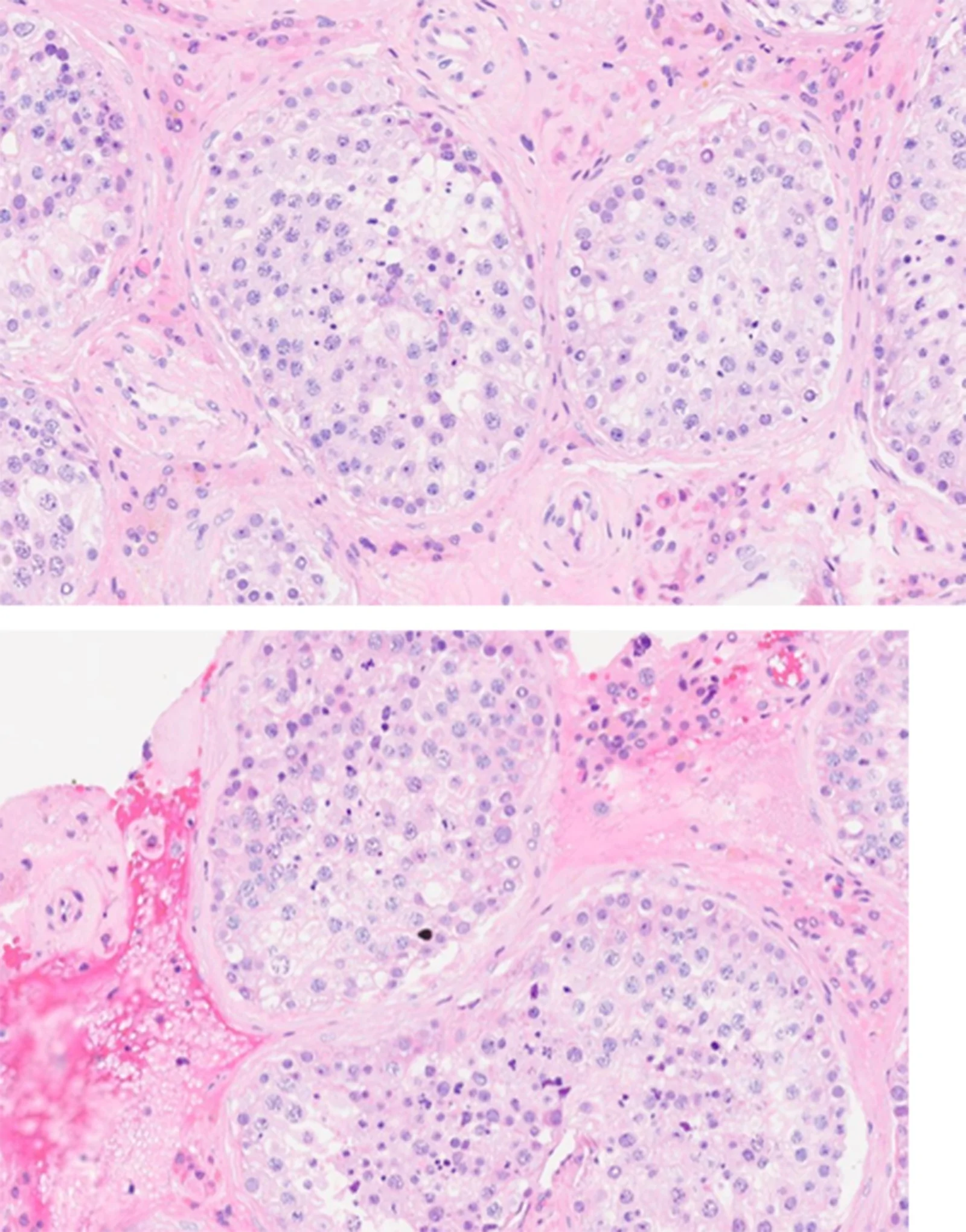
Testicular biopsy demonstrating hypospermatogenesis with partial premature detachment of spermatids and spermatocytes.

A testicular biopsy with sperm extraction was performed, yielding one immotile sperm per five fields, which was cryopreserved. Histological analysis revealed hypospermatogenesis, with spermatogenesis initiated from a substantial number of spermatogonia and completed in most tubules, but with a reduced number of mature spermatids. Partial premature detachment of spermatocytes and round spermatids was observed, with evidence of apoptosis, particularly in the latter (Fig. 2).

## Treatment

Following genetic counseling, the couple opted for assisted reproduction using the cryopreserved sperm, with preimplantation genetic (PGT-M) testing for the identified variant. Seven oocytes were injected with immotile sperm, resulting in fertilization of three oocytes.

## Outcome and follow-up

Treatment was discontinued due to lack of embryonic development. The couple subsequently decided to pursue assisted reproduction with donor sperm. No further follow-up data is available.

## Discussion

This case describes a patient with infertility and testicular microlithiasis as the sole manifestation of CNC, in the absence of other typical phenotypic features. The diagnosis of CNC was supported by the presence of testicular microlithiasis, a family history of phenotypic traits associated with the syndrome, and a pathogenic *PRKAR1A* variant [5]. Testicular microlithiasis, which has been associated with various benign and malignant testicular conditions, is also a recognized finding in CNC [6].

Ultrasound typically shows multiple, nonshadowing hyperechoic foci measuring 1-3 mm within the testicular parenchyma [7], which correspond histologically to microcalcifications [8]. Reported prevalence varies according to study design. Prospective series in men with various testicular or scrotal symptoms demonstrated rates of 12.8% in a cohort of 1538 patients [9] and 18.1% in a cohort of 1078 [10]. In a prevalence study involving asymptomatic men aged 18 to 35 years old, testicular microlithiasis was identified in 5.6% of participants [11]. The etiology is heterogeneous and may include cryptorchidism [12]. Increased prevalence has also been reported in genetic syndromes such as Klinefelter [13] and Down syndrome [14], compared with the general population. Testicular microlithiasis should be distinguished from the calcifications seen in large-cell calcifying Sertoli cell tumors, which can also occur in CNC. In our patient, the microcalcifications corresponded to true microlithiasis on biopsy and not to calcifications associated with large-cell calcifying Sertoli cell tumors.

Confirmed azoospermia can also be a feature of CNC. Reproductive function in men with CNC has been infrequently investigated [15]. PKA is a serine-threonine kinase involved in metabolism, proliferation, differentiation, and apoptosis regulation [16], and both *PRKAR1A* and PKA play a central role in spermatogenesis.

Burton et al reported severe fertility impairment in male mice heterozygous for *PRKAR1A*. Their sperm showed morphological abnormalities and reduced counts, attributed to increased PKA catalytic activity in pachytene germ cells. In men with CNC carrying *PRKAR1A* mutations, a similar phenotype was observed: in a series of seven patients, semen samples showed a high proportion of morphologically abnormal sperm (head and tail defects, immature forms). Three were azoospermic or oligospermic, and sperm defects were described in both unrelated probands and affected family members [17]. These findings suggest that *PRKAR1A* haploinsufficiency results in impaired spermatogenesis, abnormal sperm morphology, and reduced fertility, which may limit transmission of the variant.

Wieacker et al also described a patient with azoospermia, tall stature, cutaneous manifestations, cardiac myxoma, and pituitary microadenoma. Testicular biopsy revealed spermatogenic arrest at the spermatid stage in most tubules [18]. A recent report also described severe oligoasthenozoospermia in a man with CNC and a novel PRKAR1A deletion, supporting a direct role for PRKAR1A haploinsufficiency in male infertility [19]. Together, these data support that spermatogenic impairment may be a common manifestation of CNC, leading to infertility.

In our patient, histological findings were consistent with those described in mice, with evidence of apoptosis and detachment at the primary spermatocyte stage [17]. Preservation of spermatogenesis at early stages explains the normal testicular volume and FSH levels. However, during intracytoplasmic sperm injection, sperm showed morphological and functional defects resulting in poor embryonic development, which may further reduce fertility and transmission of the mutation even with assisted reproduction.

The clinical diagnosis of CNC can be established in a proband with two or more main diagnostic criteria, or with one suggestive feature plus a pathogenic (or likely pathogenic) germline *PRKAR1A* variant. Penetrance exceeds 95% by age 50. CNC is inherited in an autosomal dominant manner. About 70% of cases are familial, while ∼30% result from de novo *PRKAR1A* variants [20]. If molecular testing identifies a variant in the proband, parental studies are recommended to confirm inheritance and provide accurate counseling. If neither parent carries the variant and biological parentage is confirmed, the variant is de novo or due to parental germline mosaicism.

A negative family history may be misleading, as affected relatives can go unrecognized or die before symptoms appear. Thus, evaluation of at-risk relatives with clinical assessment and molecular testing is essential. Identifying asymptomatic carriers allows early surveillance and timely treatment.

In this case, the diagnosis was suspected based on the mother's clinical features and later confirmed by positive molecular testing. The patient's siblings have a 50% risk of CNC due to the autosomal dominant inheritance pattern.

## Learning points

- Testicular microlithiasis may represent the only phenotypic manifestation of CNC and should raise suspicion in the presence of a suggestive family history.
- *PRKAR1A* mutations can impair spermatogenesis, resulting in abnormal sperm morphology, azoospermia or oligospermia, and reduced fertility.
- Infertility in men with azoospermia or oligospermia, when accompanied by testicular microlithiasis or family history of CNC, may represent an underrecognized feature of the syndrome and should prompt genetic evaluation.
- Early identification of at-risk relatives through genetic testing enables timely surveillance and management of Carney complex–related complications.

## Contributors

All authors made individual contributions to authorship. P.K., E.A., S.M.S., and P.R.C. were involved in the clinical evaluation of the patient, interpretation of clinical, laboratory, imaging, and histopathological findings, and therapeutic decision-making. G.R.V. performed the surgery. S.K. conducted and interpreted the genetic analysis. All authors reviewed and approved the final version and agree to be accountable for all aspects of the work.

## Data Availability

Original data generated and analyzed during this study are included in this published article.
