# Correction to: “Infertility as a possible diagnostic feature of Carney complex”

**DOI:** 10.1210/jcemcr/luag302

**Published:** 2026-10-06

In the above-named article by Knoblovits P, Abelleira E, Valzacchi GR, Suárez SM, Costanzo PR, and Kleppe S (*JCEM Case Reports*. 2026; 4(6); doi: 10.1210/jcemcr/luag065), there was an error in the abstract and in the Diagnostic Assessment section.

In the originally published article, in the abstract, a sentence reads, “Family history revealed CNC-related conditions in his mother and brother, and genetic testing confirmed a pathogenic PRKAR1A variant (c.479_480del; p.Ala160Glufs*5).

In the corrected article, the sentence reads, “Family history revealed CNC-related conditions in his mother and brother, and genetic testing confirmed a pathogenic PRKAR1A variant (c.479del; p.Ala160Glufs*5).

In the originally published article, in the Diagnostic Assessment section, a sentence reads, “Molecular testing of the patient's mother, performed by sequencing and deletion/duplication analysis of the PRKAR1A gene, identified a pathogenic variant, c.479_480del (p.Ala160Glufs*5).”

In the corrected article, the sentence reads, “Molecular testing of the patient's mother, performed by sequencing and deletion/duplication analysis of the PRKAR1A gene, identified a pathogenic variant, c.479del (p.Ala160Glufs*5).”

The article has been corrected online.

doi: 10.1210/jcemcr/luag065

